# Case of a Rupture of the Tendo Achillis

**Published:** 1787

**Authors:** John Rodbard

**Affiliations:** Surgeon at Ipswich


### IV. *Caſe of a Rupture of the Tendo Achillis. Communicated in a Letter to Dr.* Simmons *by Mr.* John Rodbard, *Surgeon at Ipſwich.*

ABOUT five years ſince, ſtepping acroſs a kennel, my foot was placed too ſhort upon a ſtone to bear my weight without ſlipping. Fearing I ſhould fall, I exerted my whole ſtrength to prevent it, and ruptured the tendo Achillis about three inches above its inſertion into the os calcis. In the courſe of my practice I had met with three ſimilar caſes, and treated them according to the directions of the late Profeſſor Monro, keeping the foot conſtantly extended, and the patient confined, until the parts were again united; and I found when they began to walk that it was with great difficulty and pain, and that a conſiderable time elapſed before they could get the heel low enough to do it with any degree of comfort, eſpecially up hill. Indeed I have always obſerved obſerved that ſuch patients walk up an aſcent with great trouble, which induced me to try if nature would not furniſh renovating matter, callus, or whatever name may be given it, to fill up the ſpace without extending the foot, and if by that means I might not be freed from the pain and difficulty of getting the heel to the ground, which my patients had complained of ſo much. The experiment ſucceeded to my wiſh; I kept my foot in its natural poſition, followed my buſineſs, walked and rode conſiderably every day, and took care only to give the ancle joint as little motion as poſſible till I found a perfect union of the tendon. I can now walk or run up an aſcent, or up ſtairs, without pain, and uſe this limb as well as the other. The leg is waſted conſiderably, but the thigh is equally fleſhy as the other. I have ſince met with another caſe, ſimilar to mine, which was treated in the ſame manner with equal ſucceſs, and in which I only kept a ſlight bandage round the ancle and foot, moiſtened with Goulard's vegeto-mineral water.

